# Understanding Genomic Instability Score in Platinum-Treated Advanced Pancreatic Ductal Adenocarcinoma

**DOI:** 10.3390/jcm15197632

**Published:** 2026-10-02

**Authors:** Justus Leinhos, Theodora Malli, Anna Gabler, Daniel Nothdurfter, Andreas Reichinger, Jonathan Burghofer, Stefan Schwarz, Johann Mandl, Georg Schreil, Alexander Andorfer, Hannes Windhager, Karl J. Aichberger, Patrick Kirchweger, Florian Primavesi, Gerald Webersinke, Holger Rumpold, Bernhard Doleschal

**Affiliations:** 1 Department of Internal Medicine I for Hematology with Stem Cell Transplantation, Hemostaseology and Medical Oncology, Ordensklinikum Linz, 4010 Linz, Austria; justus.leinhos@ordensklinikum.at or k12024822@students.jku.at or justus.leinhos@med.uni-muenchen.de (J.L.); anna.gabler@ordensklinikum.at (A.G.); daniel.nothdurfter@ordensklinikum.at (D.N.); andreas.reichinger@ordensklinikum.at or andreas.reichinger@jku.at (A.R.); hannes.windhager@ordensklinikum.at (H.W.); holger.rumpold@ordensklinikum.at (H.R.); 2 Faculty of Medicine, Johannes Kepler University Linz, 4020 Linz, Austria; jonathan.burghofer@jku.at or jonathan.burghofer@ordensklinikum.at (J.B.); patrick.kirchweger@jku.at (P.K.); gerald.webersinke@jku.at or gerald.webersinke@ordensklinikum.at (G.W.); 3 Department of Internal Medicine III, Hematology and Oncology, LMU University Hospital, 81377 Munich, Germany; 4 Laboratory for Molecular Genetic Diagnostics, Ordensklinikum Linz, 4010 Linz, Austria; theodora.malli@ordensklinikum.at (T.M.); stefan.schwarz@ordensklinikum.at (S.S.); 5 Department of Internal Medicine IV for Hematology and Medical Oncology, Hospital Wels-Grieskirchen, 4600 Wels, Austria; johann.mandl@klinikum-wegr.at; 6 Department of Internal Medicine II for Medical Oncology, State Hospital Pyhrn-Eisenwurzen, 4400 Steyr, Austria; georg.schreil@ooeg.at; 7 Department of Internal Medicine I for Hematology and Medical Oncology, Sisters of Charity Hospital Ried, 4910 Ried im Innkreis, Austria; alexander.andorfer@bhs.at; 8 Department of Internal Medicine, State Hospital Rohrbach, 4150 Rohrbach, Austria; karl.aichberger@ooeg.at; 9 Department of Surgery, Ordensklinikum Linz, 4010 Linz, Austria; florian.primavesi@ordensklinikum.at

**Keywords:** pancreatic cancer, genomic instability score, platinum chemotherapy, homologous recombination deficiency, predictive biomarker, precision oncology

## Abstract

**Background/Objectives:** First-line treatment selection for advanced (locally advanced or metastatic) pancreatic ductal adenocarcinoma (PDAC) is currently based on clinical characteristics rather than predictive biomarkers. While homologous recombination deficiency (HRD) is associated with increased sensitivity to platinum-based chemotherapy, established predictive biomarkers, such as pathogenic alterations in *BRCA1*, *BRCA2*, and *PALB2*, identify only a small subset of patients who are likely to benefit from platinum-based first-line treatment. The Genomic Instability Score (GIS) may capture broader HRD-associated genomic scarring beyond single-gene testing and support biomarker-guided platinum selection. However, the clinical utility of the GIS in PDAC remains uncertain, and no PDAC-specific GIS cutoff has yet been established. **Methods:** In this retrospective multicenter cohort, 127 patients with advanced PDAC underwent TruSight Oncology 500 HRD testing between 2022 and 2025. GIS was evaluable in 99 patients; 80 received first-line therapy. The primary endpoint was time to failure of the first-line strategy (TFS). An exploratory candidate GIS cutoff for platinum sensitivity was identified using maximally selected log-rank statistics, and survival was analyzed using Kaplan–Meier estimates and Cox regression. **Results:** In the first-line platinum cohort, an exploratory GIS threshold of 39 showed the greatest discrimination for treatment failure-free survival (TFS) within this cohort. Patients with GIS ≥ 39 had significantly longer TFS than those with GIS < 39 (9.9 vs. 5.9 months; HR 0.33, 95% CI 0.13–0.80; *p* = 0.015), and this association remained significant after multivariable adjustment. Among individual genes, *BRCA2*-mutated tumors exhibited significantly higher GIS than *BRCA2*-wild-type tumors (median 54 vs. 19; FDR-adjusted *p* = 0.002), although 30% of GIS-high tumors lacked *BRCA1/2* mutations. Combined stratification by GIS and first-line treatment demonstrated a stepwise improvement in overall survival, with median OS of 8.2 months in non-platinum/GIS < 39 patients, 15.1 months in platinum/GIS < 39 patients, and 30.7 months in platinum/GIS ≥ 39 patients (*p* = 0.002). **Conclusions:** GIS may identify patients with advanced PDAC who experience favorable outcomes during first-line platinum-based chemotherapy. The observed association between higher GIS and improved treatment outcomes supports further investigation of GIS as a biomarker for treatment stratification in advanced PDAC and warrants prospective validation.

## 1. Introduction

Advanced pancreatic cancer remains one of the most lethal malignancies, with increasing incidence and persistently poor survival [1,2]. Selecting the most appropriate first-line treatment for patients with advanced (locally advanced or metastatic) pancreatic ductal adenocarcinoma (PDAC) remains a clinical challenge. Although gemcitabine plus nab-paclitaxel (Gem-nabP) and platinum-containing therapies, including FOLFIRINOX and NALIRIFOX, are widely used first-line regimens, they differ substantially in efficacy, toxicity, and tolerability [1,3,4,5,6]. FOLFIRINOX or NALIRIFOX may provide superior efficacy in selected patients but are associated with considerable toxicity, limiting their use in broader clinical populations [1,4,5]. Currently, treatment selection between Gem-nabP and platinum-based regimens is guided primarily by clinical factors rather than robust predictive biomarkers [3,6].

Accurate histological diagnosis is a prerequisite for molecular biomarker-guided treatment selection in pancreatic cancer. PDAC must be reliably distinguished from rarer pancreatic neoplasms with distinct biology and management, such as pancreatic acinar cell carcinoma, pancreatic neuroendocrine tumors (pNET) or solid pseudopapillary neoplasm. Imaging-based differentiation from PDAC remains an active area of investigation for these entities [7,8,9,10]. Beyond diagnostic accuracy, early detection of PDAC itself remains critical, as smaller, earlier-stage tumors are associated with substantially better resectability and survival than larger, more advanced tumors. This underscores the importance of pancreatic cancer screening in at-risk individuals [11]. Combining nuclear medicine techniques with other imaging modalities, such as MRI or PET-CT, may further improve the accuracy of early PDAC detection [12].

Homologous recombination repair (HRR) deficiency has been associated with increased sensitivity to platinum-based chemotherapy across several malignancies, including ovarian, breast, pancreatic, and other gastrointestinal cancers [3,13,14,15,16]. Pathogenic alterations in key HRR genes can impair DNA repair, resulting in homologous recombination deficiency (HRD). In pancreatic ductal adenocarcinoma (PDAC), pathogenic variants in *BRCA1*, *BRCA2*, and *PALB2* are the most common HRR alterations; however, they account for only 5–10% of cases [2,3,13]. HRD gives rise to characteristic patterns of genomic scarring, including loss of heterozygosity (LOH), telomeric allelic imbalance (TAI), and large-scale state transitions (LST) [17,18,19]. These genomic scar signatures are integrated into the Genomic Instability Score (GIS), which serves as a surrogate marker of HRD [18,19]. While GIS-based HRD assessment has demonstrated predictive value in several tumor types, its clinical utility in advanced PDAC remains insufficiently defined [3,13,14,15,19].

To date, no PDAC-specific GIS cutoff has been established, and the predictive value of GIS for platinum sensitivity remains uncertain. We therefore aimed to evaluate the GIS as a candidate biomarker associated with outcomes during first-line platinum-based chemotherapy in advanced PDAC and to explore a GIS threshold associated with treatment outcomes in this setting [3,19,20].

## 2. Materials and Methods

We conducted a multicenter, real-world study including patients with advanced PDAC treated in 5 centers in Austria between 2022 and 2025. All patients underwent next-generation sequencing (NGS) using the TruSight Oncology 500 HRD assay (Illumina Inc., San Diego, CA, USA). The TruSight Oncology 500 HRD assay is a hybrid-capture-based NGS panel covering 523 cancer-related DNA genes (1.94 Mb) and 55 RNA genes, enabling the detection of single-nucleotide variants (SNVs), insertions/deletions (indels), copy-number variations (CNVs), gene fusions, tumor mutational burden (TMB), microsatellite instability (MSI), and homologous recombination deficiency (HRD) [21,22]. Sequencing was performed on an Illumina NextSeq 550 platform according to the manufacturer’s instructions [22]. Primary bioinformatic processing was conducted on an in-house DRAGEN server using the TruSight Oncology 500 Enrichment App (Illumina Inc., San Diego, CA, USA), followed by tertiary variant annotation in VarSeq (Golden Helix, Inc., Bozeman, MT, USA). The clinical relevance of detected variants was assessed using ClinVar (National Center for Biotechnology Information, Bethesda, MD, USA), COSMIC (Wellcome Sanger Institute, Hinxton, UK), VarSome (Saphetor SA, Lausanne, Switzerland), and, where required, Alamut Visual Plus (SOPHiA GENETICS SA, Rolle, Switzerland). Only pathogenic or likely pathogenic variants, classified according to ACMG/AMP criteria, were included in gene-specific analyses [23,24,25,26,27,28]. More than 95% of target regions achieved a sequencing depth of at least 100 non-duplicated reads, ensuring high analytical performance across SNVs, indels, CNVs, and gene fusions [21,22]. A minimum tumor cell content of 20% was required for TMB and MSI assessment, whereas at least 30% was required for reliable GIS calculation [22]. Tumors with an MSI score < 20% were classified as microsatellite stable (MSS), whereas those with an MSI score ≥ 20% were classified as MSI-high [22]. Genomic scars comprise somatic structural alterations resulting from defective homologous recombination repair, including loss of heterozygosity (LOH), telomeric allelic imbalance (TAI), and large-scale state transitions (LST). These three genomic scar signatures are integrated into the Genomic Instability Score (GIS), a surrogate marker of homologous recombination deficiency. GIS was calculated using the TruSight Oncology 500 HRD assay based on a proprietary algorithm developed in collaboration with Myriad Genetics [22].

HRR gene alterations were categorized into core and non-core HRR genes based on the strength and consistency of their established association with homologous recombination deficiency (HRD). *BRCA1*, *BRCA2*, and *PALB2* were classified as core HRR genes, reflecting their well-established biological role in homologous recombination and the clinical relevance of pathogenic alterations in these genes. *ATM*, *BAP1*, *BARD1*, *BLM*, *BRIP1*, *CHEK2*, *FAM175A*, *FANCA*, *FANCC*, *NBN*, *RAD50*, *RAD51*, *RAD51C*, and *RTEL1* were classified as non-core HRR genes. For these genes, the association between pathogenic alterations, a functional HRD phenotype, and sensitivity to DNA-damaging therapies is less consistently established and may depend on tumor type and biological context [3].

All samples consisted of formalin-fixed, paraffin-embedded (FFPE) tissue obtained from diagnostic biopsies or repeat biopsies at disease progression. Samples were collected from seven Upper Austrian clinics and centrally processed at the Upper Austrian Cancer Center. The study was conducted under the supervision of the Upper Austrian Cancer Center and included patients from Ordensklinikum Linz, Hospital Wels-Grieskirchen, Sisters of Charity Hospital Ried, State Hospital Rohrbach and State Hospital Pyhrn-Eisenwurzen Steyr.

Molecular profiling, including NGS-based GIS assessment, was performed independently of first-line treatment selection and did not inform the choice of initial therapy. First-line treatment was determined according to established clinical criteria, including ECOG performance status and comorbidities (Supplementary Figure S1). Molecular profiling was primarily undertaken to characterize tumors and identify potential options for subsequent or targeted therapies, with GIS obtained as an additional readout of the NGS analysis. Sequencing was completed before initiation of first-line treatment in 52 of 80 patients (65.0%), whereas in 28 patients (35.0%), sequencing results became available after treatment had already commenced.

The primary objective of this study was to evaluate the potential role of the Genomic Instability Score (GIS) as a biomarker associated with clinical benefit from platinum-based chemotherapy in advanced PDAC. The primary endpoint was time to failure of the first-line strategy (TFS). Secondary endpoints included overall survival (OS) and radiological response, categorized as complete response (CR), partial response (PR), stable disease (SD), or progressive disease (PD).

TFS was defined as the interval from initiation of first-line treatment to the first occurrence of addition or substitution of an agent not included in the initial treatment strategy, disease progression during ongoing first-line treatment, disease progression without subsequent therapy, or death from any cause [29,30]. Patients without an event at the last follow-up were censored at the date of the most recent clinical contact. Tumor response was assessed according to the Response Evaluation Criteria in Solid Tumors (RECIST) version 1.1 [31].

Clinicopathologic data included demographic, clinical, histopathological, molecular, and treatment-related variables, which were extracted from institutional records and electronic medical records. All data were anonymized prior to analysis.

Descriptive statistics were used to summarize patient demographics, clinicopathologic characteristics, and genomic data. Continuous variables are presented as median and interquartile range (IQR) and were compared using the Mann–Whitney U test or Kruskal–Wallis test, as appropriate. Categorical variables were compared using the chi-square test or Fisher’s exact test. Associations between GIS and clinical outcomes were explored using nonparametric tests and receiver operating characteristic (ROC) analysis.

The optimal GIS cutoff for platinum sensitivity was identified using maximally selected log-rank statistics. To account for the multiple testing inherent in evaluating several candidate cutoffs, a cutoff-search-adjusted *p*-value was estimated using a conditional Monte Carlo approximation based on 99,999 permutations. Internal validation was performed using 2000 bootstrap samples. Within each bootstrap sample, the complete cutoff-selection procedure was repeated and the cutoff stability was assessed using the bootstrap distribution of selected cutoffs. Survival analyses were performed using Kaplan–Meier estimates and Cox proportional hazards regression models. Patients were additionally stratified into three exploratory groups according to a calculated GIS cut-off and first-line treatment type. The proportional hazards assumption was evaluated using Schoenfeld residuals. Exploratory spline-based Cox regression models were used to visualize the continuous relationship between GIS and the hazard of treatment failure. Associations between GIS and specific gene alterations were assessed using Wilcoxon rank-sum tests, with adjustment for multiple comparisons by the Benjamini–Hochberg false discovery rate (FDR) procedure.

All statistical tests were two-sided, and *p* < 0.05 was considered statistically significant. Statistical analyses were performed using SPSS (version 29.0; IBM Corp., Armonk, NY, USA) and R (version 4.3.3; R Foundation for Statistical Computing, Vienna, Austria) with the packages *dplyr*, *survival*, *ggplot2*, *survminer*, and *gtsummary*. Analyses were performed using R version 4.5.3 with the following packages: dplyr version 1.2.1, survival version 3.8-6, ggplot2 version 4.0.1, survminer version 0.5.2, and gtsummary version 2.5.1.

The study was approved by the local ethics committee of Upper Austria on 11 June 2026 (reference number: 1069/2026). All procedures were performed in accordance with relevant laws, institutional guidelines, and the Declaration of Helsinki. Written informed consent was obtained from all patients, and all data were anonymized in compliance with institutional data protection regulations and the General Data Protection Regulation (GDPR, EU 2016/679) [32].

This was a retrospective, non-interventional study based on electronic medical records, including clinical, molecular, treatment, and survival data up to October 2025. No experimental interventions or additional sampling were performed beyond standard diagnostic and therapeutic procedures. All patient identifiers were removed prior to analysis. Disease stages were classified according to the eighth edition of the TNM classification system. The study was conducted in accordance with the REMARK guidelines [33,34]. This real-world data study was reported in accordance with the ESMO Guidance for Reporting Oncology Real-World Evidence (ESMO-GROW). The completed ESMO-GROW checklist is provided as Supplementary File S1 [35,36].

## 3. Results

**Figure 1:**
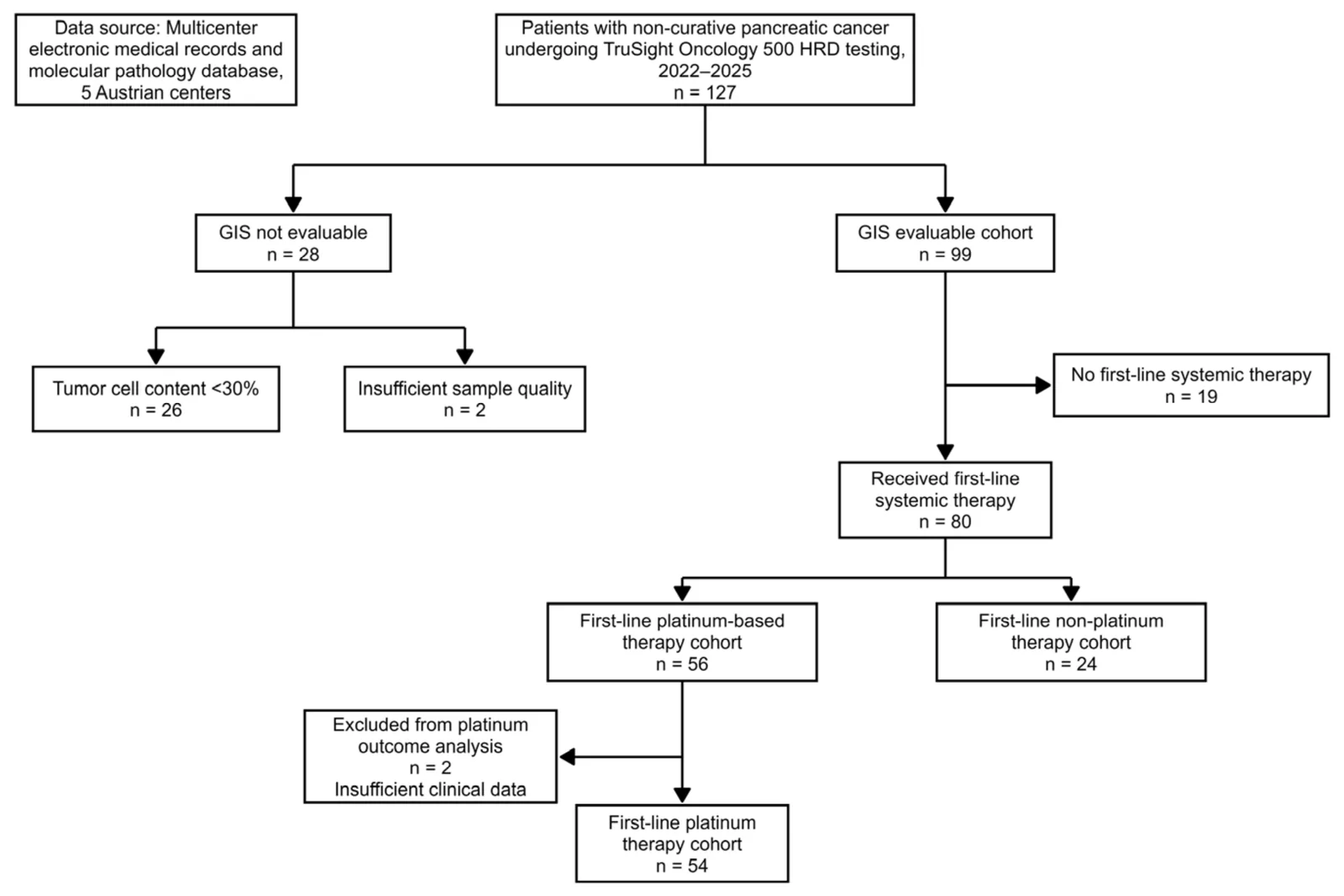
Patient selection and stratification by GIS and first-line treatment.

We included 127 patients with advanced PDAC who underwent NGS-based molecular profiling. In 26 cases, insufficient tumor content (<30%) precluded GIS calculation, and in two additional cases, overall sample quality was insufficient. Consequently, GIS was evaluable in 99 patients. Of these, 80 received first-line systemic therapy (56 platinum based and 24 gemcitabine plus nab-paclitaxel). Two platinum-treated patients had insufficient clinical data for outcome analyses (Figure 1).

The median age at treatment initiation was 64.0 years (IQR, 58.0–71.0). Patients receiving non-platinum therapy were older than those treated with platinum-containing regimens (72.0 vs. 62.0 years) and had poorer ECOG performance status (*p* = 0.006). Tumor mutational burden and tumor cell content were comparable between groups, whereas the median GIS was higher in the platinum-treated cohort than in the non-platinum cohort (20.0 vs. 17.0; *p* = 0.012). Sex, ethnicity, MSI status, and the frequencies of *BRCA1/2* and other HRR gene alterations were comparable between treatment groups; all tumors were microsatellite stable. Liver and lymph node metastases were the most common metastatic sites, followed by lung metastases and peritoneal carcinomatosis, with no statistically significant differences between treatment groups. Second-line treatment was administered more frequently in the platinum group than in the non-platinum group (66.1% vs. 37.5%; *p* = 0.026), whereas the proportions of patients receiving third-, fourth-, and fifth-line therapies were comparable (Table 1).

Treatment discontinuation due to adverse drug reactions occurred in 13.0% of patients receiving first-line platinum-based therapy and 8.3% of those receiving non-platinum-based therapy, with no significant difference between groups (Fisher’s exact *p* = 0.713).

**Figure 2:**
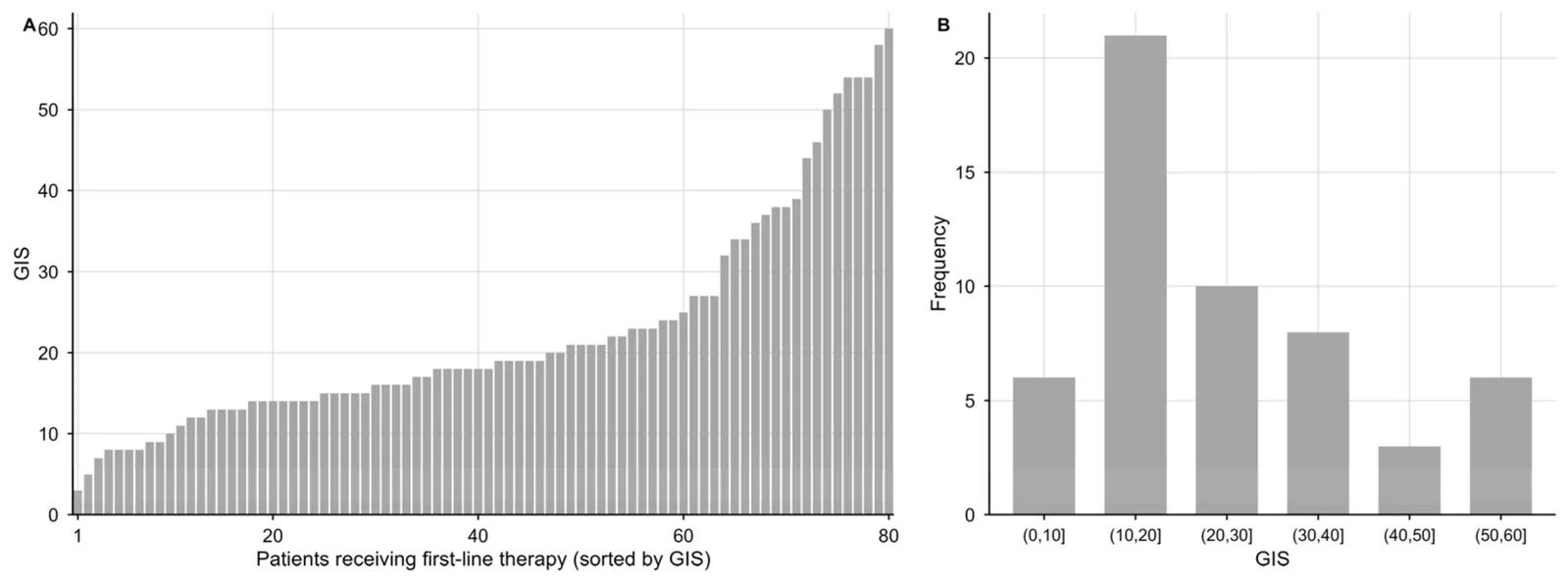
Distribution of Genomic Instability Score (GIS) values. (**A**) GIS values ranked in ascending order across patients receiving first-line therapy. (**B**) Binned distribution of GIS values in patients receiving first-line platinum-containing therapy.

Among patients receiving first-line systemic therapy, the median GIS was higher in the platinum-treated cohort than in the non-platinum cohort (20.0 [IQR, 14.0–35.5; range, 5–60] vs. 17.0 [IQR, 12.3–19.8]; *p* = 0.012). Most GIS values in the first-line platinum cohort clustered within the low-to-intermediate range, particularly between 10 and 30, whereas values > 40 were observed only in a small subset of tumors (Figure 2).

**Figure 3:**
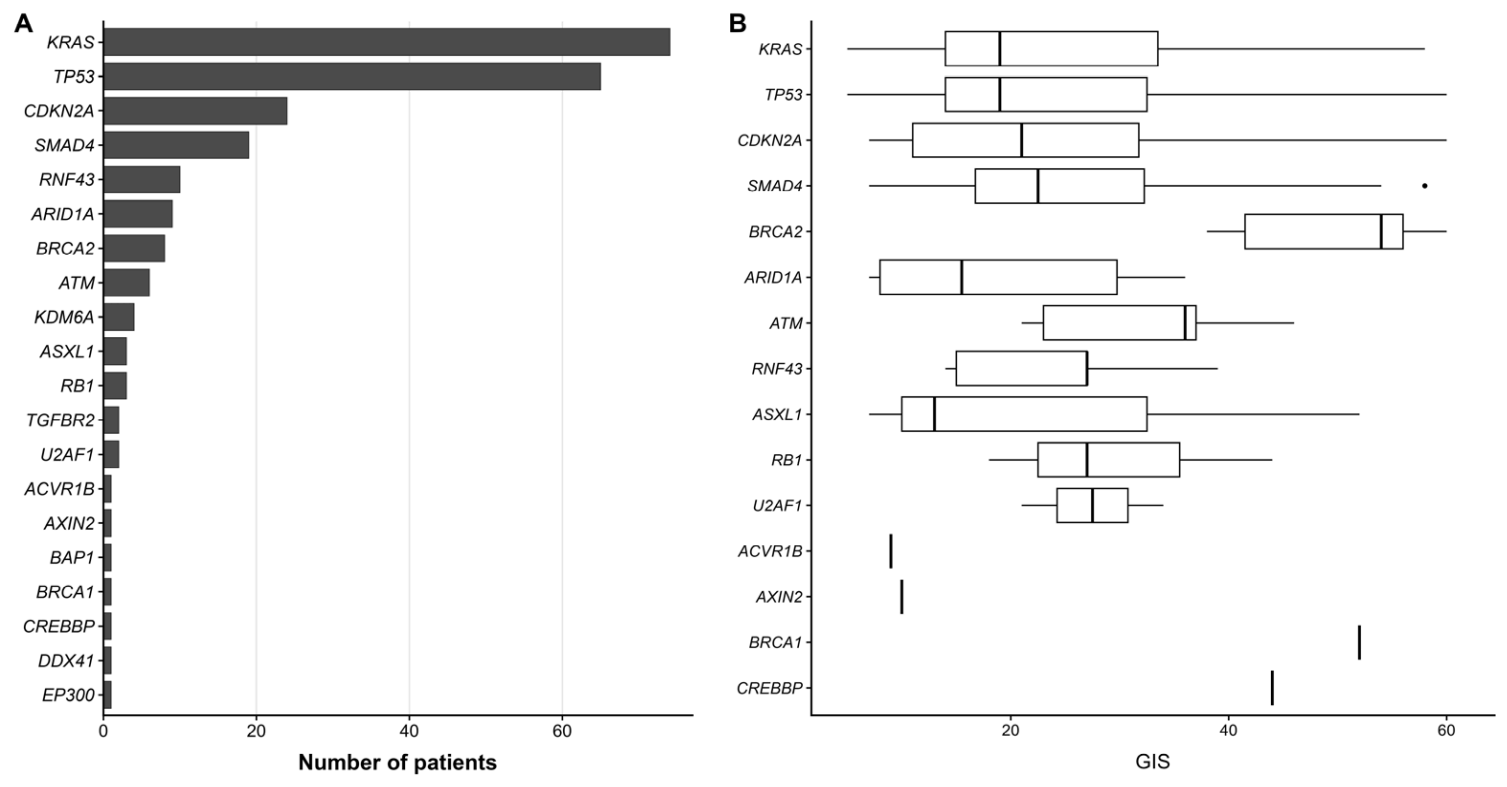
Mutational landscape and distribution of Genomic Instability Scores according to recurrent gene alterations. (**A**) Bar plot showing the 20 most frequently altered genes in the first-line treatment cohort, ordered by decreasing alteration frequency. (**B**) Boxplots showing the distribution of Genomic Instability Score (GIS) values among tumors harboring mutations in the 15 most frequently altered genes in the first-line platinum outcome cohort (*n* = 54). Boxes represent the interquartile range, the vertical line within each box indicates the median, whiskers extend to the most extreme values within 1.5 times the interquartile range, and dots denote outliers. Genes altered in a single tumor are shown as a single vertical line.

NGS analysis of the 80 patients with evaluable GIS in the first-line treatment cohort identified recurrent pathogenic or likely pathogenic alterations, predominantly in *KRAS* (92.5%) and *TP53* (81.3%), with less frequent alterations in *CDKN2A* (30.0%) and *SMAD4* (23.8%). The distribution of the 20 most frequently altered genes is shown in Figure 3A. In the first-line platinum cohort (*n* = 54), GIS distributions were compared between mutated and wild-type tumors for the 15 most frequently altered genes (Figure 3B). Among these, *BRCA2* alterations showed the strongest association with genomic instability. *BRCA2*-mutated tumors had substantially higher GIS values than *BRCA2* wild-type tumors (median, 54 vs. 19; IQR, 41.5–56.0 vs. 14.0–27.0; nominal *p* < 0.001; FDR-adjusted *p* = 0.002). Although GIS values varied across tumors harboring other recurrent alterations, including *KRAS*, *TP53*, *CDKN2A*, *SMAD4*, *ARID1A*, and *ATM*, none remained significantly associated with GIS after correction for multiple testing.

**Figure 4:**
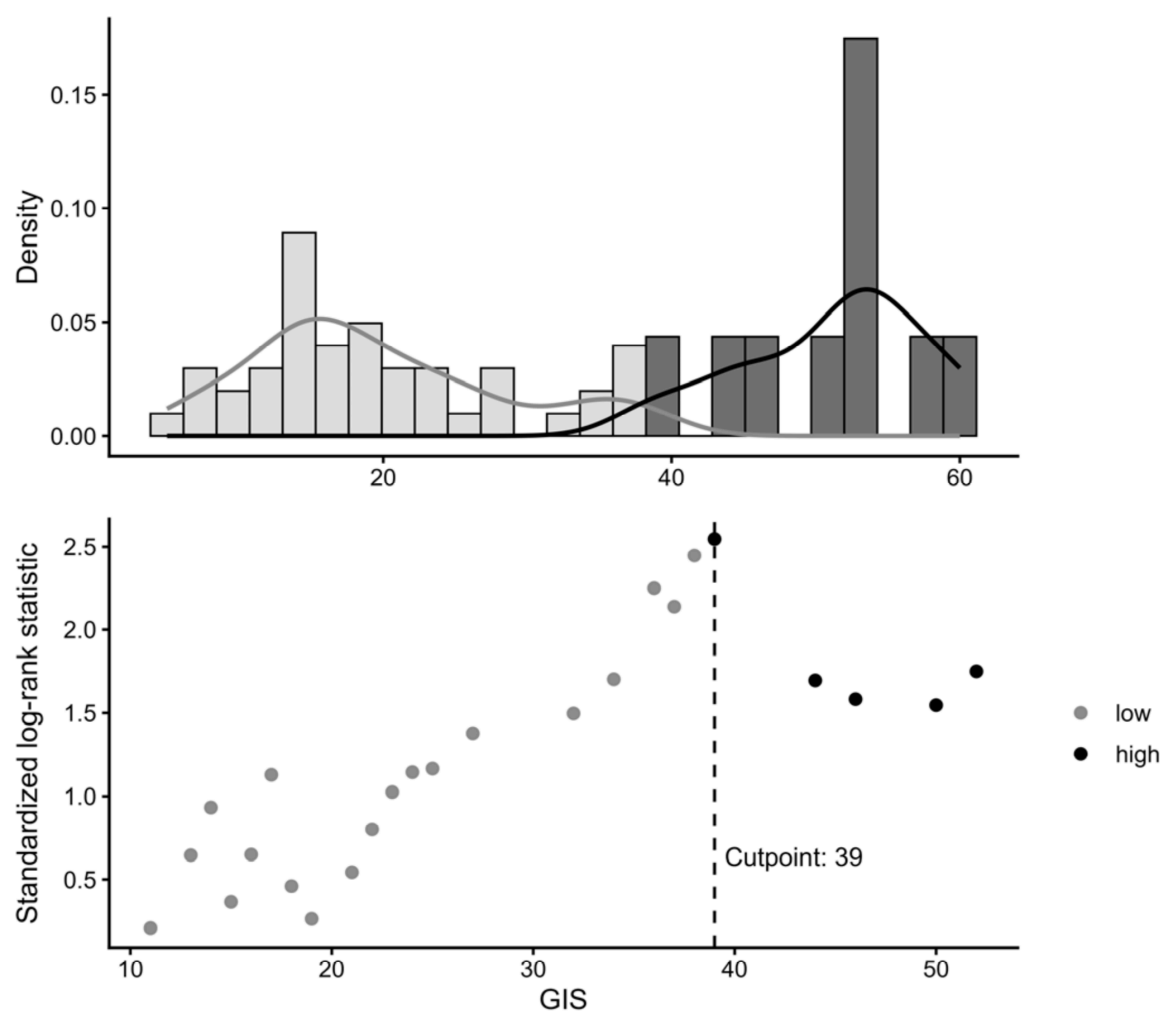
GIS cutoff determination. Distribution of Genomic Instability Score (GIS) values and maximally selected log-rank analysis for time to failure of the first-line strategy (TFS) in the platinum cohort, identifying GIS ≥ 39 as the optimal cutoff (nominal log-rank *p* = 0.011, Monte Carlo selection-adjusted *p* = 0.027). Upper panel: histogram of GIS values with kernel density curves; light grey bars and the grey line represent tumors with GIS < 39, and dark grey bars and the black line represent tumors with GIS ≥ 39. Lower panel: standardized log-rank statistic for each candidate cutoff; grey and black dots indicate candidate values below and at or above the selected cutoff, respectively, and the vertical dashed line marks the selected cutoff of 39.

**Figure 5:**
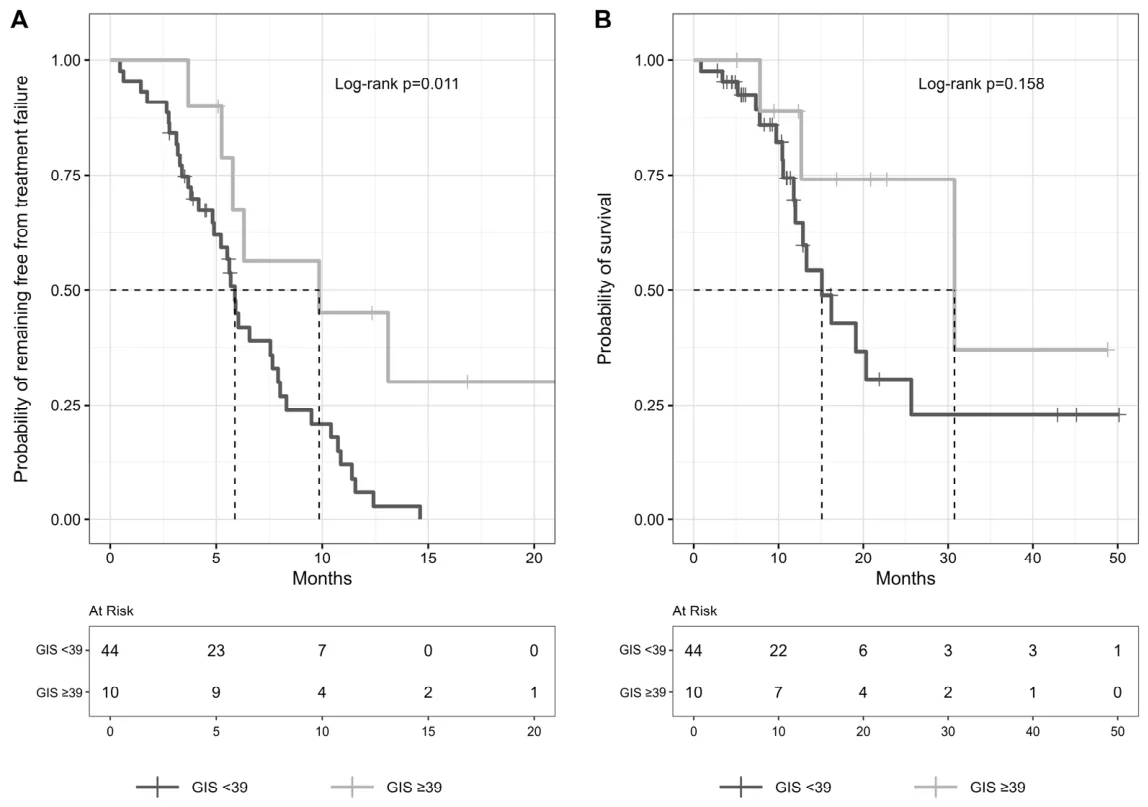
TFS and OS analyses according to the GIS cutoff of 39. (**A**) Kaplan–Meier analysis of TFS in the first-line platinum cohort, stratified by GIS < 39 versus GIS ≥ 39 (median 5.9 vs. 9.9 months; multivariable adjusted HR 0.27, 95% CI 0.09–0.80, *p* = 0.018). (**B**) Kaplan–Meier analysis of OS using the same cutoff (median 15.1 vs. 30.7 months; multivariable adjusted HR 0.10, 95% CI 0.02–0.55, *p* = 0.008). Dashed lines indicate median TFS and OS, and tick marks denote censored observations. Numbers at risk are shown below each plot.

To explore a clinically relevant GIS threshold associated with outcomes during platinum-based therapy, TFS was selected as the endpoint for threshold determination. In the platinum cohort, maximally selected log-rank analysis identified GIS ≥ 39 as the optimal candidate threshold associated with improved outcome following platinum-containing therapy (Figure 4). Patients with GIS ≥ 39 (GIS-high tumors) had a longer median TFS than patients with GIS < 39 (9.9 vs. 5.9 months; standardized log-rank statistic 2.546, χ^2^ = 6.484, nominal log-rank *p* = 0.011 and cutoff-search-adjusted *p* = 0.027; Figure 5A). Cox regression further supported this finding, showing a reduced risk of treatment failure in the GIS ≥ 39 group compared with the GIS < 39 group (HR 0.33, 95% CI 0.13–0.80; *p* = 0.015). In exploratory spline-based Cox modeling, increasing GIS values above the median were associated with a progressively lower estimated hazard of first-line strategy failure (Figure S2).

Bootstrap analysis demonstrated that the cutoff distribution was concentrated around the candidate threshold of 39. Across 2000 bootstrap resamples, the 25th percentile, median and 75th percentile of the selected cutoffs were 36, 39 and 39, respectively. The exact cutoff of 39 was selected in 35.3% of bootstrap samples, whole 64.9% of the selected cutoffs were between 31 and 41 (Figure S3). The GIS-high subgroup (GIS ≥ 39; *n* = 10) was predominantly composed of tumors harboring *BRCA1/2* mutations (70.0%, *n* = 7). One tumor (10.0%) harbored a pathogenic HRR gene alteration other than *BRCA1/2*, while two tumors (20.0%) were without an identifiable pathogenic HRR gene alteration. Detailed characteristics of the GIS-high subgroup are summarized in Table S1. Baseline characteristics were largely balanced between the GIS ≥ 39 group versus the GIS < 39 group in the first-line platinum outcome cohort (Table S2). GIS-high tumors had a slightly higher tumor mutational burden (5.9 vs. 3.1 mut/Mb; *p* < 0.001), higher tumor cell content (60.0% vs. 50.0%; *p* = 0.049), and more frequent *BRCA1/2* mutations (70.0% vs. 2.3%; *p* < 0.001). When considering any HRR gene alteration (*BRCA1/2* or other core/non-core HRR genes combined), this difference remained pronounced (80.0% vs. 13.6%; Fisher’s exact *p* < 0.001). Age, ECOG performance status, sex, ethnicity, and metastatic-site distribution did not differ significantly between groups. Subsequent treatment exposure was comparable between groups, with a median number of treatment lines of 1.5 treatment lines in the GIS ≥ 39 group and 2.0 in the GIS < 39 group (*p* = 0.214). The proportion of patients receiving at least two treatment lines (50.0% vs. 72.7%, *p* = 0.257) and the distribution of exactly two versus three or more treatment lines among these patients were also comparable (*p* = 1.000; Table S2). In the complete first-line platinum cohort (*n* = 54; 43 TFS events), GIS ≥ 39 was associated with a lower risk of TFS failure in a multivariable Cox model adjusted for age, ECOG performance status, number of metastatic sites, and number of treatment lines (adjusted HR 0.27, 95% CI 0.09–0.80; *p* = 0.018).

In an exploratory multivariable Cox model adjusted for overall HRR mutation status, ECOG performance status, number of metastatic sites, and number of treatment lines, GIS ≥ 39 remained independently associated with a lower risk of TFS failure (adjusted HR 0.24, 95% CI 0.07–0.84; *p* = 0.026), whereas overall HRR mutation status was not independently associated with TFS (HR 1.32, 95% CI 0.52–3.39; *p* = 0.560). No significant GIS-by-HRR interaction was observed (interaction HR 0.39, 95% CI 0.05–2.85; *p* = 0.353; likelihood-ratio *p* = 0.371). In a multivariable model additionally adjusted for tumor cell content, GIS ≥ 39 remained independently associated with a lower risk of TFS failure (adjusted HR 0.23, 95% CI 0.08–0.67; *p* = 0.007), and a GIS × tumor cell content interaction term was not statistically significant (*p* = 0.154).

For OS, patients with GIS ≥ 39 had a numerically longer median survival than those with GIS < 39 (30.7 vs. 15.1 months), without a statistically significant difference in the unadjusted analysis (log-rank *p* = 0.158; HR 0.42, 95% CI 0.12–1.45; Cox *p* = 0.171; Figure 5B). After adjustment for ECOG performance status and number of treatment lines in a multivariable Cox model (*n*= 54; 20 OS events), GIS ≥ 39 was associated with a lower hazard of death (adjusted HR 0.10, 95% CI 0.02–0.55; *p* = 0.008). In a corresponding model additionally adjusted for tumor cell content, GIS ≥ 39 remained independently associated with a lower risk of death (adjusted HR 0.08, 95% CI 0.02–0.44; *p* = 0.003), and a GIS × tumor cell content interaction term was not statistically significant (*p* = 0.219).

When the GIS threshold of 42, established in other tumor entities, was applied within the first-line platinum cohort, no statistically significant separation was observed for either TFS or OS (TFS log-rank *p* = 0.090 and OS log-rank *p* = 0.299; Figure S4), and this remained non-significant after multivariable adjustment for age, ECOG performance status, number of metastatic sites, and number of treatment lines for TFS (adjusted HR 0.55, 95% CI 0.22–1.37, *p* = 0.198), and for ECOG performance status and number of treatment lines for OS (adjusted HR 0.38, 95% CI 0.10–1.40, *p* = 0.145).

**Figure 6:**
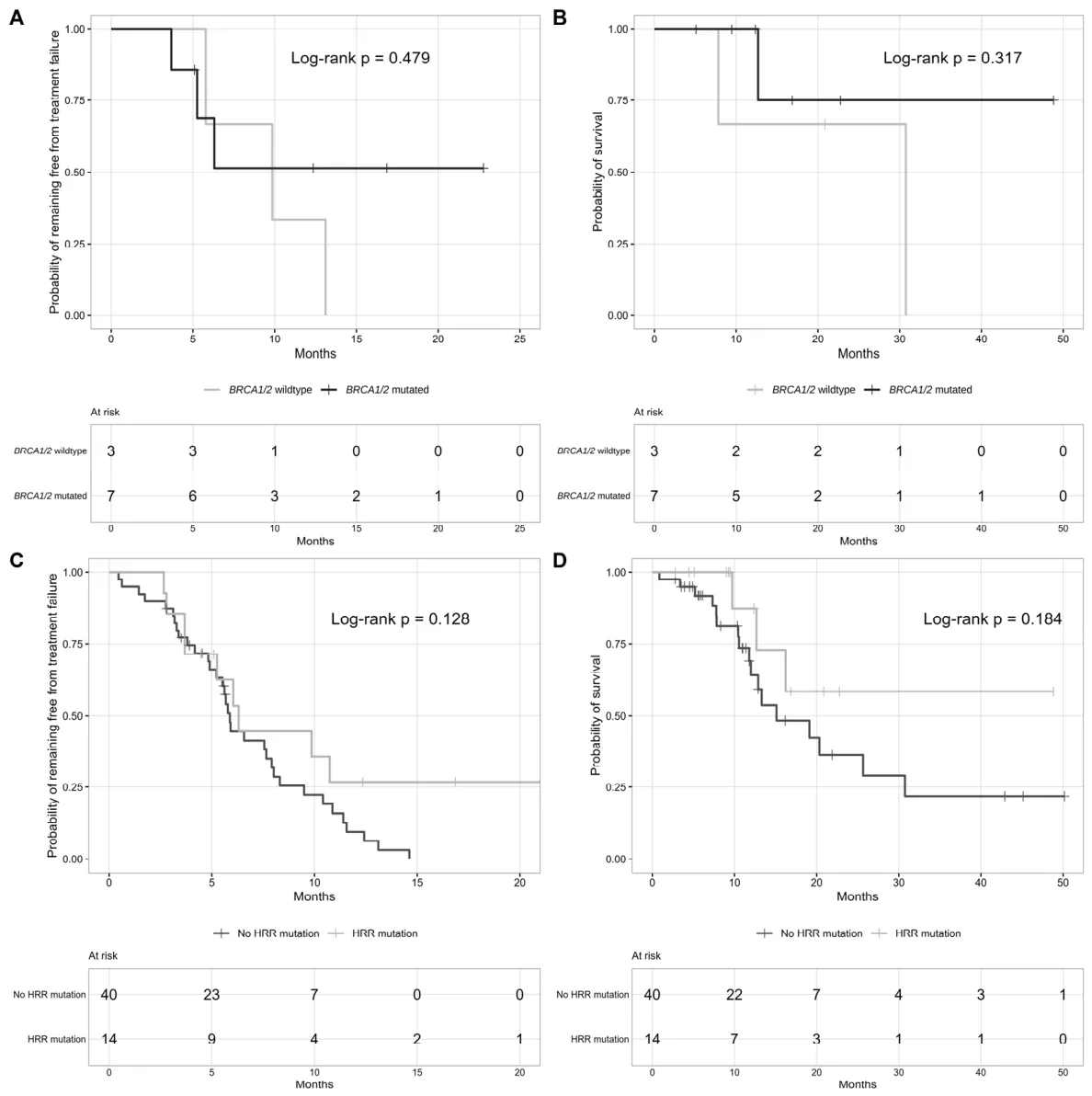
Treatment failure-free survival and overall survival according to *BRCA1/2* and homologous recombination repair mutation status. Kaplan–Meier analyses of (**A**) treatment failure-free survival (TFS) and (**B**) overall survival (OS) according to *BRCA1/2* mutation status among patients with GIS ≥ 39 (*n* = 10). (**C**) TFS and (**D**) OS according to overall homologous recombination repair (HRR) mutation status in the first-line platinum cohort (*n* = 54).

Within the GIS ≥ 39 subgroup, no significant difference was observed between *BRCA1/2*-mutated and *BRCA1/2*-wild-type tumors for TFS (log-rank *p* = 0.479; HR 0.56, 95% CI 0.11–2.81) or OS (log-rank *p* = 0.317; HR 0.31, 95% CI 0.03–3.50) (Figure 6A,B). Likewise, when the entire platinum-treated cohort was stratified by the presence of any HRR gene mutation, no significant differences were observed for TFS (log-rank *p* = 0.128) or OS (log-rank *p* = 0.184) (Figure 6C,D).

Radiological response outcomes were numerically higher in the GIS ≥ 39 subgroup within the platinum-treated cohort. The objective response rate was 70.0% in patients with GIS ≥ 39 compared with 56.8% in those with GIS < 39 (*p* = 0.501), while the corresponding disease control rates were 90.0% and 75.0%, respectively (*p* = 0.426; Figure S5). Among patients achieving an objective response (CR or PR), a trend toward longer duration of response was observed in the GIS ≥ 39 group (median 9.9 vs. 5.7 months; HR 0.37, 95% CI 0.12–1.10; log-rank *p* = 0.064).

To further explore the combined effect of GIS status and platinum exposure, patients were stratified into three groups: GIS < 39 with non-platinum therapy, GIS < 39 with platinum-based therapy, and GIS ≥ 39 with platinum-based therapy. No patients receiving non-platinum therapy had a GIS ≥ 39; therefore, a fourth non-platinum/GIS ≥ 39 subgroup could not be analyzed. This exploratory analysis revealed a stepwise increase in median TFS across the three available groups, from 3.8 months in the non-platinum/GIS < 39 group to 5.9 months in the platinum/GIS < 39 group and 9.9 months in the platinum/GIS ≥ 39 group (log-rank *p* = 0.058). A more pronounced separation was observed for OS, with median survival increasing from 8.2 months in the non-platinum/GIS < 39 group to 15.1 months in the platinum/GIS < 39 group and 30.7 months in the platinum/GIS ≥ 39 group (log-rank *p* = 0.002; Figure S6).

Detailed results of exploratory subgroup and response analyses are provided in the Supplementary Materials.

## 4. Discussion

This study addresses the unmet need for molecular biomarkers that can refine first-line treatment selection in advanced PDAC. Rather than being defined by a single genomic alteration, HRD may be better viewed as a composite biological phenotype that can be captured by genomic scar–based measures. Our findings are consistent with growing evidence that HRD and genomic instability are associated with increased platinum sensitivity across several tumor types. In ovarian and triple-negative breast cancer, genomic scar-based HRD scores have been linked to response to platinum-containing therapy [14,15,37]. Our study extends these observations by evaluating the GIS in a multicenter, real-world cohort of patients with advanced PDAC receiving first-line treatment.

*BRCA1* and *BRCA2* remain the most established gene-based predictors of platinum sensitivity within this framework. In our cohort, *BRCA2*-mutated tumors had substantially higher GIS values than *BRCA2*-wild-type tumors, and *BRCA1/2* alterations accounted for 70.0% of the GIS-high subgroup. These observations are consistent with the established association between *BRCA1/2* alterations, genomic instability, and benefit from DNA-damaging treatment in PDAC [2,3,13]. *BRCA* status therefore provides a biologically specific marker of HRD, whereas genomic scar assessment may extend the identification of this phenotype beyond *BRCA*-defined tumors.

Beyond *BRCA1/2*, pathogenic alterations in other HRR genes have also been associated with impaired homologous recombination and an HRD phenotype. However, the clinical predictive value of broadly defined HRR-mutated groups has been inconsistent across pancreatic and other gastrointestinal malignancies [2,3,13,16,29]. In our platinum-treated cohort, the presence of any HRR gene mutation was not significantly associated with TFS or OS. This finding does not exclude a potential role for individual HRR alterations but it suggests that the presence of a HRR mutation alone may not reliably represent functional HRD. Indeed, genomic scar-based HRD assessment such as GIS is thought to capture instability phenotypes extending beyond discrete HRR gene mutations, including non-core alterations, epigenetic *BRCA1* promoter silencing, or other structural genomic scarring not reflected in gene-level mutation calling [38].

GIS integrates loss of heterozygosity, telomeric allelic imbalance, and large-scale state transitions into a cumulative measure of chromosomal instability resulting from deficient homologous recombination repair [18,19]. Previous PDAC studies have evaluated different HRD scores, mutational signatures, machine-learning approaches, and whole-genome classifiers and have reported varying associations with platinum sensitivity and clinical outcomes [3,4,13,39,40]. Collectively, these studies support the potential relevance of genomic scar-based HRD assessment but have not established a pancreas-specific GIS threshold for patients with advanced PDAC receiving first-line therapy.

In our cohort, GIS ≥ 39 emerged as the threshold that best separated patients according to the durability of first-line platinum-based therapy. Patients with GIS ≥ 39 experienced significantly longer TFS and a lower risk of treatment failure, while exploratory analyses of OS showed a consistent direction of association. Combined stratification by platinum exposure and GIS further revealed a stepwise gradient in OS, supporting a potential relationship between genomic instability, platinum exposure, and clinical outcome in this setting. By contrast, the established GIS cutoff of ≥42, which originated in other tumor types and was subsequently applied in PDAC studies, did not significantly discriminate TFS or OS in our cohort, including after multivariable adjustment [13,40]. These findings suggest that the clinically informative range of GIS in advanced PDAC may differ from thresholds established in other disease contexts. Although GIS ≥ 39 provided greater separation in the present cohort, its potential relevance as a PDAC-specific threshold requires validation in independent cohorts.

If prospectively validated, GIS could be incorporated into established molecular diagnostic workflows and may help refine first-line treatment selection in advanced PDAC. The present findings support GIS as a complementary stratification marker alongside *BRCA1/2* status, clinical fitness, comorbidities, and expected treatment tolerability. Such an integrated approach may improve the identification of patients most likely to derive durable benefit from platinum-based therapy without relying solely on either a single gene alteration or an isolated genomic score.

Several limitations should be considered. The retrospective, non-randomized design limits causal inference, and no patient with GIS ≥ 39 received non-platinum first-line therapy, precluding a direct comparison of the relative benefit of platinum versus non-platinum treatment within the GIS-high subgroup. Accordingly, our findings should be regarded as hypothesis-generating rather than as evidence of a treatment-predictive effect of GIS. The GIS cutoff of 39 was derived and evaluated within the same cohort and has not undergone external validation. Although bootstrap resampling suggested reasonable internal stability (Figure S3), prospective validation in independent PDAC cohorts is required.

The GIS-high and gene-defined subgroups were small, and treatment regimens were heterogeneous. Moreover, a targeted panel does not provide the resolution of whole-genome mutational signatures or a direct functional assessment of homologous recombination. Future studies should therefore compare prespecified GIS thresholds and formally assess the interaction between GIS and platinum exposure, ideally alongside complementary genomic and functional measures of HRD.

Finally, several practical limitations may affect clinical implementation. The interval from diagnosis to availability of NGS-based GIS results may be too long to inform first-line treatment decisions without delaying treatment initiation, particularly at centers without rapid in-house sequencing capacity. In addition, reliable GIS calculation requires a minimum tumor cell content of 30%, a threshold that may not be met by all diagnostic samples. Although tumor cellularity correlated with GIS values in our cohort, we found no evidence that tumor cell content modified the association between GIS and clinical outcomes. These considerations should be taken into account when evaluating GIS as a biomarker for first-line treatment selection.

## 5. Conclusions

This study identified an association between GIS and clinical outcomes in patients with advanced PDAC receiving first-line platinum-based chemotherapy. GIS was associated with clinically relevant outcomes and may provide information beyond BRCA mutation status. Further prospective studies are needed to determine whether GIS may help identify patients who derive greater benefit from platinum-based therapy. Independent validation is warranted to further define the clinical relevance of GIS in advanced PDAC.

## Figures and Tables

**Table 1:** Baseline characteristics. Baseline clinical, molecular, and metastatic characteristics of patients with advanced PDAC, stratified by platinum-based versus non-platinum therapy.

Variable	Total (*N* = 80)	Non-Platinum (*n* = 24)	Platinum (*n* = 56)	*p*-Value *
Continuous variables, median (IQR)				
Age at therapy initiation, years	64.0 (58.0–71.0)	72.0 (63.5–77.0)	62.0 (54.8–66.0)	—
Tumor mutational burden (mut/Mb)	3.6 (2.4–5.4)	3.9 (2.4–4.8)	3.6 (2.4–5.5)	—
Tumor cell content (%)	50 (35–60)	50 (37.5–70.0)	50.0 (35–60)	—
Genomic instability score (GIS)	18.0 (14.0–24.0)	17.0 (12.3–19.8)	20.0 (14.0–35.5)	0.012
Categorical variables, *n* (%)				
ECOG performance status (advanced disease)				
– 0	39 (48.8%)	7 (29.2%)	32 (57.1%)	
– 1	33 (41.3%)	11 (45.8%)	22 (39.3%)	
– 2	8 (10.0%)	6 (25.0%)	2 (3.6%)	0.006
Sex				
– Female	34 (42.5%)	11 (45.8%)	23 (41.1%)	
– Male	46 (57.5%)	13 (54.2%)	33 (58.9%)	0.693
Ethnicity				
– Caucasian	68 (85.0%)	21 (87.5%)	47 (83.9%)	
– Other	12 (15.0%)	3 (12.5%)	9 (16.1%)	0.682
MSI status				
– MSS	80 (100%)	24 (100%)	56 (100%)	—
HRR genes				
*BRCA1/2* mutation, *n* (%)	9 (11.3%)	1 (4.2%)	8 (14.3%)	0.266
Other HRR gene mutation, *n* (%)	7 (8.8%)	1 (4.2%)	6 (10.7%)	0.668
Metastatic sites				
Lymph node metastases	41 (51.3%)	14 (58.3%)	27 (48.2%)	0.407
Lung metastases	19 (23.8%)	9 (37.5%)	10 (17.9%)	0.058
Bone metastases	4 (5.0%)	1 (4.2%)	3 (5.4%)	0.823
Liver metastases	48 (60.0%)	15 (62.5%)	33 (58.9%)	0.765
Peritoneal carcinomatosis	18 (22.5%)	8 (33.3%)	10 (17.9%)	0.129
Other metastases	5 (6.3%)	2 (8.3%)	3 (5.4%)	0.614
Treatment lines received, *n* (%) ^†^				
First-line treatment	80 (100%)	24 (100%)	56 (100%)	—
Second-line treatment	46 (57.5%)	9 (37.5%)	37 (66.1%)	0.026
Third-line treatment	20 (25.0%)	4 (16.7%)	16 (28.6%)	0.399
Fourth-line treatment	8 (10.0%)	2 (8.3%)	6 (10.7%)	1.000
Fifth-line treatment	5 (6.3%)	0 (0%)	5 (8.9%)	0.315

* *p*-values refer to comparisons between the platinum and non-platinum groups. ^†^ Percentages for individual platinum agents are calculated among patients receiving a platinum-containing regimen in the respective treatment line. Abbreviations: ECOG, Eastern Cooperative Oncology Group; GIS, Genomic Instability Score; HRR, homologous recombination repair; IQR, interquartile range; MSI, microsatellite instability; MSS, microsatellite stable; mut/Mb, mutations per megabase.

## Data Availability

The data underlying this study are not publicly available due to privacy and ethical restrictions concerning patient-level clinical and molecular data. De-identified data may be made available from the corresponding author upon reasonable request and subject to institutional and ethical approval. The analysis code is available from the corresponding author upon reasonable request.

## Supplementary Materials

The following supporting information can be downloaded at https://www.mdpi.com/article/10.3390/jcm15197632/s1, Figure S1: Workflow for molecular profiling and first-line treatment selection; Figure S2: Functional relationship between GIS and the risk of treatment failure under platinum-based therapy; Figure S3: Bootstrap stability of GIS cutoff for TFS and OS; Figure S4: TFS and OS analyses according to the GIS cutoff of 42; Figure S5: Radiological response according to GIS status in the first-line platinum cohort; Figure S6: Three-group analyses of TFS and OS according to GIS status and platinum exposure; Table S1: Patient characteristics of the GIS-high subgroup; Table S2: Baseline characteristics and subsequent treatment exposure in the first-line platinum outcome cohort according to Genomic Instability Score status; Supplementary File S1: Completed ESMO-GROW checklist.

## Abbreviations

The following abbreviations are used in this manuscript: *BRCA1/2*, breast cancer gene 1 and breast cancer gene 2; CI, confidence interval; CNV, copy number variation; CR, complete response; DDR, DNA damage response; ECOG, Eastern Cooperative Oncology Group; FDR, false discovery rate; FFPE, formalin-fixed paraffin-embedded; GDPR, General Data Protection Regulation; GIS, Genomic Instability Score; HR, hazard ratio; HRD, homologous recombination deficiency; HRR, homologous recombination repair; indel, insertion or deletion; IQR, interquartile range; LOH, loss of heterozygosity; LST, large-scale state transition; MSI, microsatellite instability; MSS, microsatellite stable; NGS, next-generation sequencing; OS, overall survival; *PALB2*, partner and localizer of *BRCA2*; PD, progressive disease; PR, partial response; RECIST, Response Evaluation Criteria in Solid Tumors; ROC, receiver operating characteristic; SD, stable disease; SNV, single nucleotide variant; TAI, telomeric allelic imbalance; TCC, tumor cell content; TMB, tumor mutational burden; TSO500, TruSight Oncology 500; TFS, time to failure of the first-line strategy; VAF, variant allele frequency.
