# Supplementary material for: Understanding Genomic Instability Score in Platinum-Treated Advanced Pancreatic Ductal Adenocarcinoma
Source: J Clin Med. 2026 Oct 2;15(19):7632. doi: 10.3390/jcm15197632 (PMC13666186; doi:10.3390/jcm15197632)
Supplement: Supplementary file 1 [file jcm-15-07632-s001.zip › jcm-4534918-supplementary.pdf]

Understanding Genomic Instability Score in Platinum-Treated Advanced Pancreatic Ductal Adenocarcinoma

Justus Leinhos [1,2,3] Theodora Malli [4], Anna Gabler [1], Daniel Nothdurfter [1], Andreas Reichinger [1,2], Jonathan Burghofer [2,4], Stefan Schwarz [4], Johann Mandl [5], Georg Schreil [6], Alexander Andorfer [7], Hannes Windhager [1], Karl J. Aichberger [8], Patrick Kirchweger [2,9], Florian Primavesi [9], Gerald Webersinke [2,4], Holger Rumpold [1] and Bernhard Doleschal [1,2,*]

* Correspondence: bernhard.doleschal@ordensklinikum.at or bernhard.doleschal@jku.at

Supplementary Materials

Figure S1. Workflow for molecular profiling and first-line treatment selection.
Figure S2. Functional relationship between GIS and risk of treatment failure under platinum-based therapy.
Figure S3. Bootstrap stability of GIS cutoff for TFS and OS.
Figure S4. TFS and OS analyses according to the GIS cutoff of 42.
Figure S5. Radiological response according to GIS status in the first-line platinum cohort.
Figure S6. Three-group analyses of TFS and OS according to GIS status and platinum exposure.
Table S1. Patient characteristics of the GIS-high subgroup.
Table S2. Baseline characteristics and subsequent treatment exposure in the first-line platinum outcome cohort according to Genomic Instability Score status.
Supplementary File S1. Completed ESMO-GROW checklist.

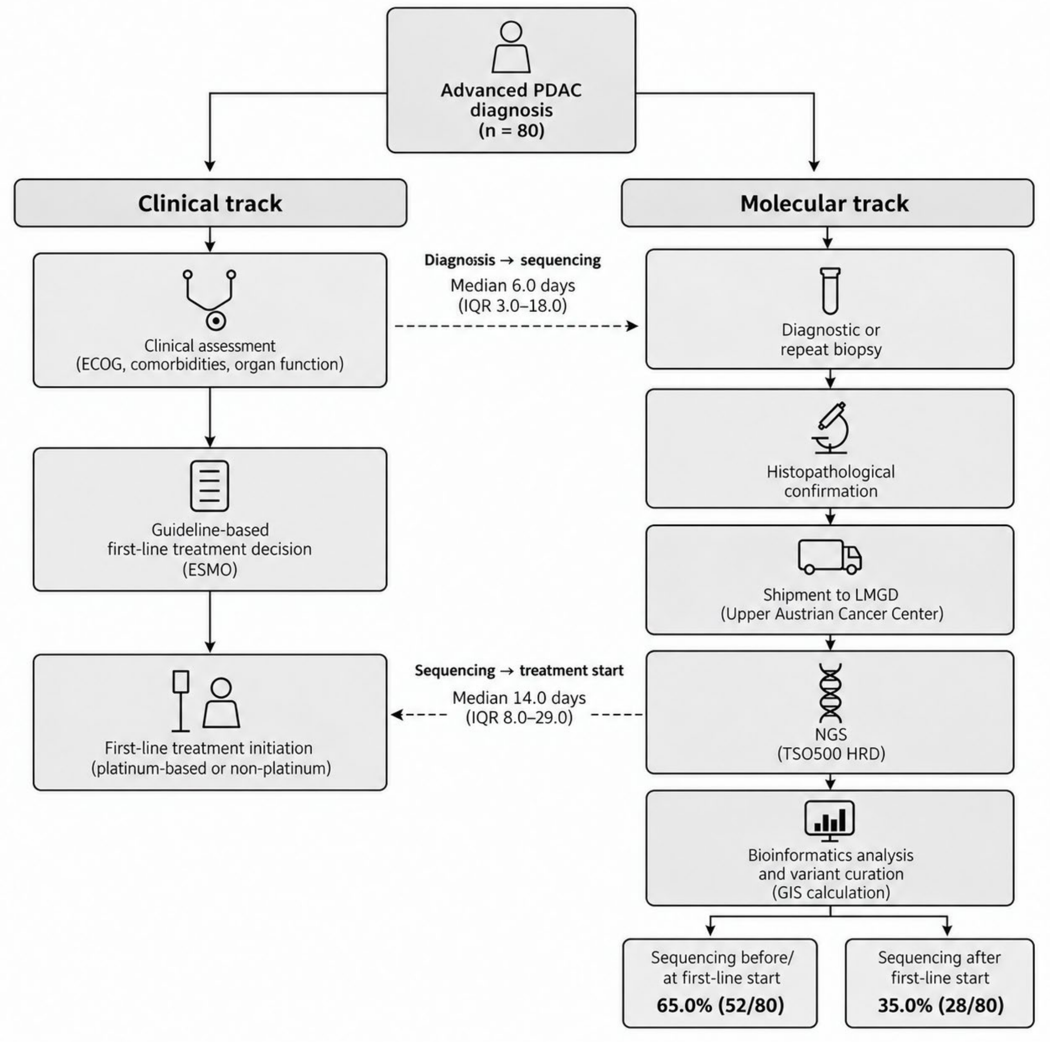

Figure S1. Workflow for molecular profiling and first-line treatment selection. The molecular pathology workflow (biopsy, histopathological confirmation, sample shipment to the LMGD, NGS/TSO500 HRD sequencing, bioinformatic analysis, and GIS calculation) proceeded in parallel with the clinical workflow (clinical assessment, guideline-based first-line treatment selection, and treatment initiation). GIS results were not used to guide first-line treatment selection. Sequencing was performed before initiation of first-line treatment in 52 of 80 patients (65.0%), with a median interval of 6.0 days (IQR, 3.0–18.0) from diagnosis to sequencing and 14.0 days (IQR, 8.0–29.0) from sequencing to treatment initiation. In the remaining 28 patients (35.0%), sequencing was performed after first-line treatment had already been initiated.

Abbreviations: ECOG, Eastern Cooperative Oncology Group; ESMO, European Society for Medical Oncology; GIS, Genomic Instability Score; HRD, homologous recombination deficiency; IQR, interquartile range; LMGD, Laboratory for Molecular Genetic Diagnostics, Upper Austrian Cancer Center; NGS, next-generation sequencing; PDAC, pancreatic ductal adenocarcinoma; TSO500, TruSight Oncology 500.

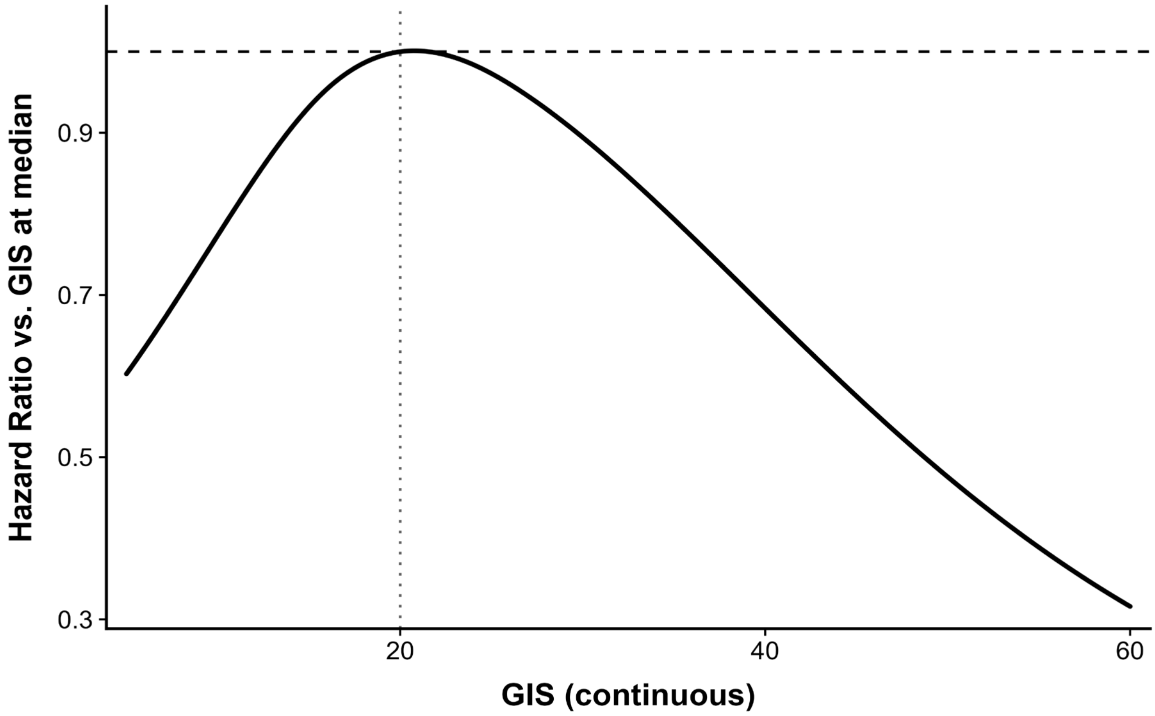

Figure S2. Functional relationship between GIS and risk of treatment failure under platinum-based therapy. Spline-based Cox regression illustrating the continuous relationship between GIS and the hazard of treatment failure in patients receiving platinum-based therapy. Higher GIS values were associated with a lower estimated hazard of treatment failure, supporting the directionality of the cutoff-based analysis.

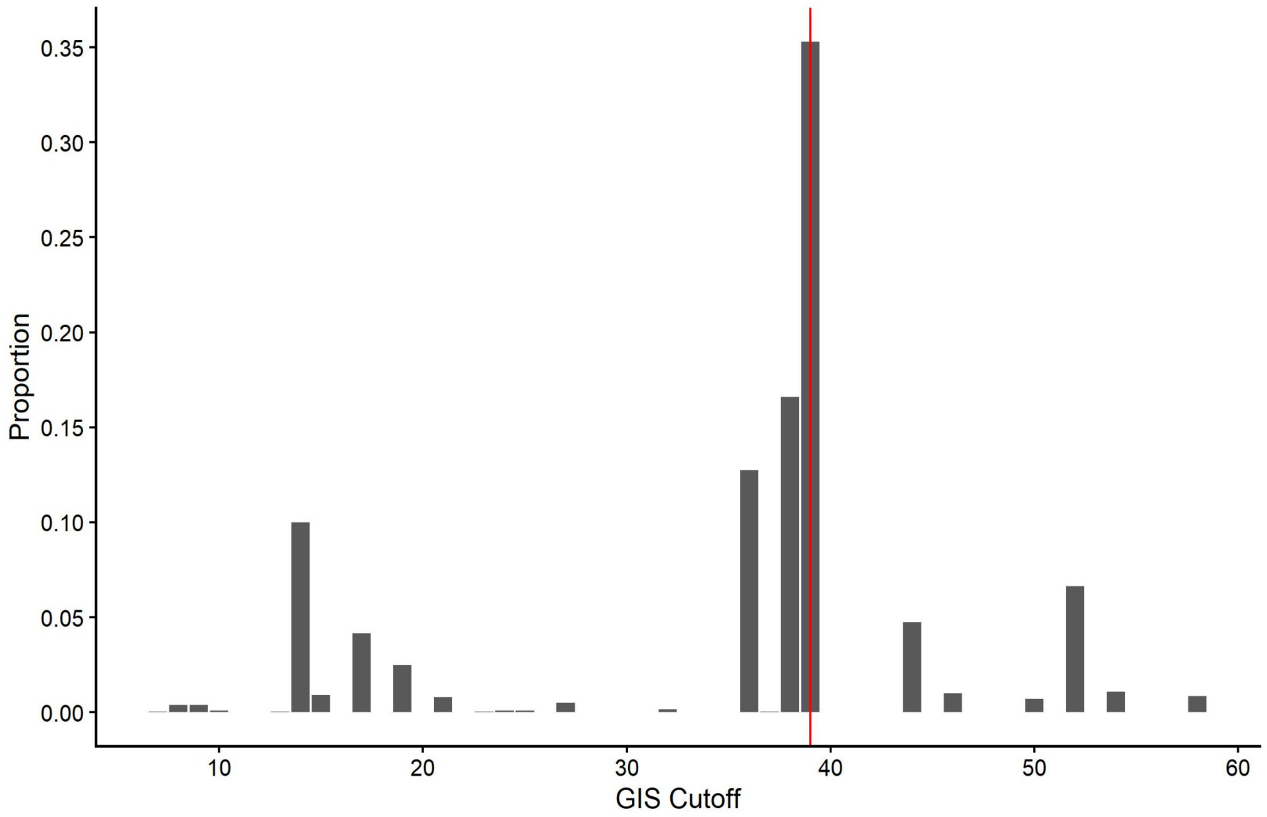

Figure S3. Bootstrap stability of the GIS cutoff for TFS. Proportional distribution of the optimal GIS cutoffs selected across 2000 bootstrap resamples. Maximally selected log-rank analysis identified GIS ≥39 as the optimal candidate threshold (M = 2.984; cutoff-search-adjusted conditional Monte Carlo p = 0.027). The 25th percentile, median, and 75th percentile of the bootstrap-selected cutoffs were 36, 39, and 39, respectively. The apparent Harrell C-index was 0.574; after correction for a mean bootstrap-estimated optimism of 0.029, the optimism-corrected C-index was 0.546.

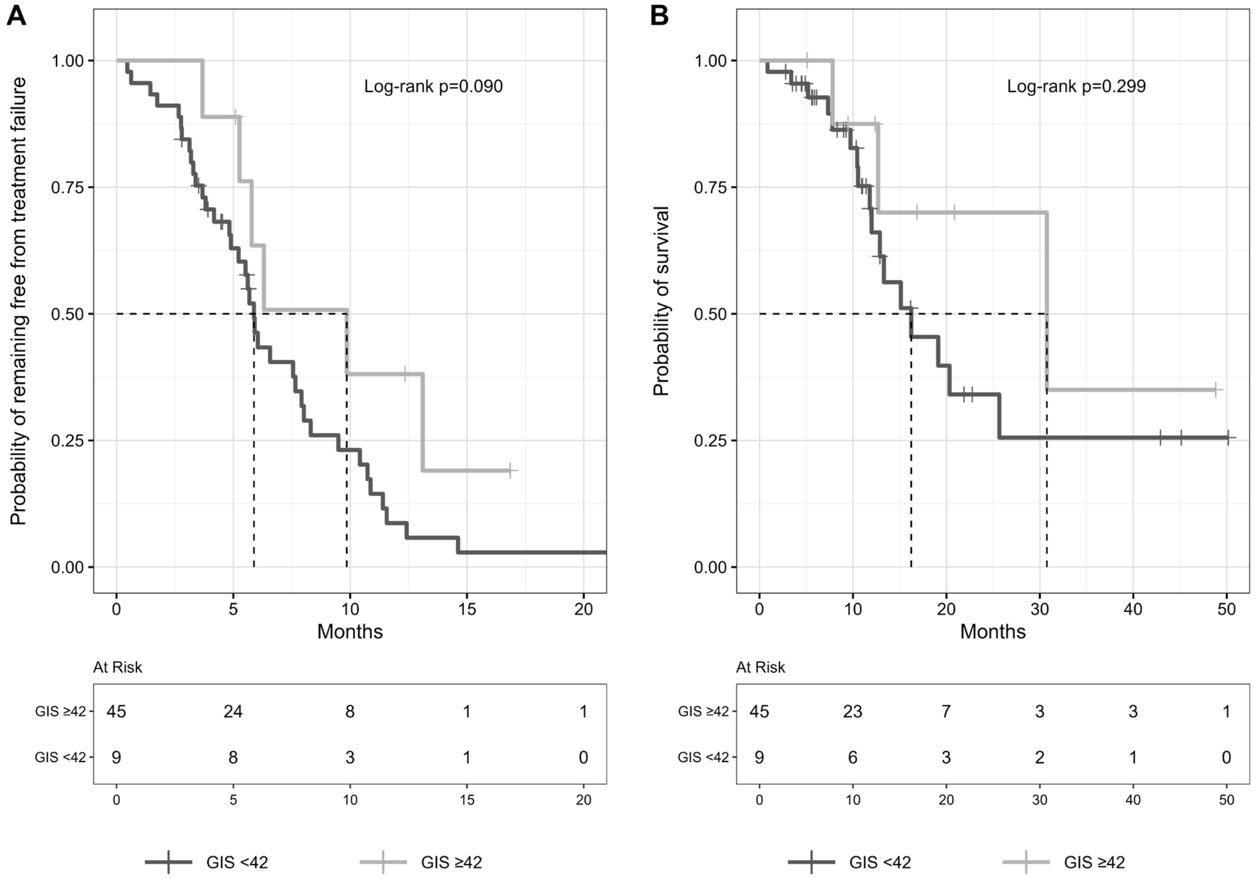

Figure S4. TFS and OS analyses according to the GIS cutoff of 42. (A) Kaplan–Meier analysis of TFS in the first-line platinum cohort, stratified by GIS <42 versus GIS ≥42 (median 5.9 vs. 9.9 months; multivariable adjusted HR 0.55, 95% CI 0.22–1.37, p = 0.198). (B) Kaplan–Meier analysis of OS using the same cutoff (median 16.2 vs. 30.7 months; multivariable adjusted HR 0.38, 95% CI 0.10–1.40, p = 0.145).

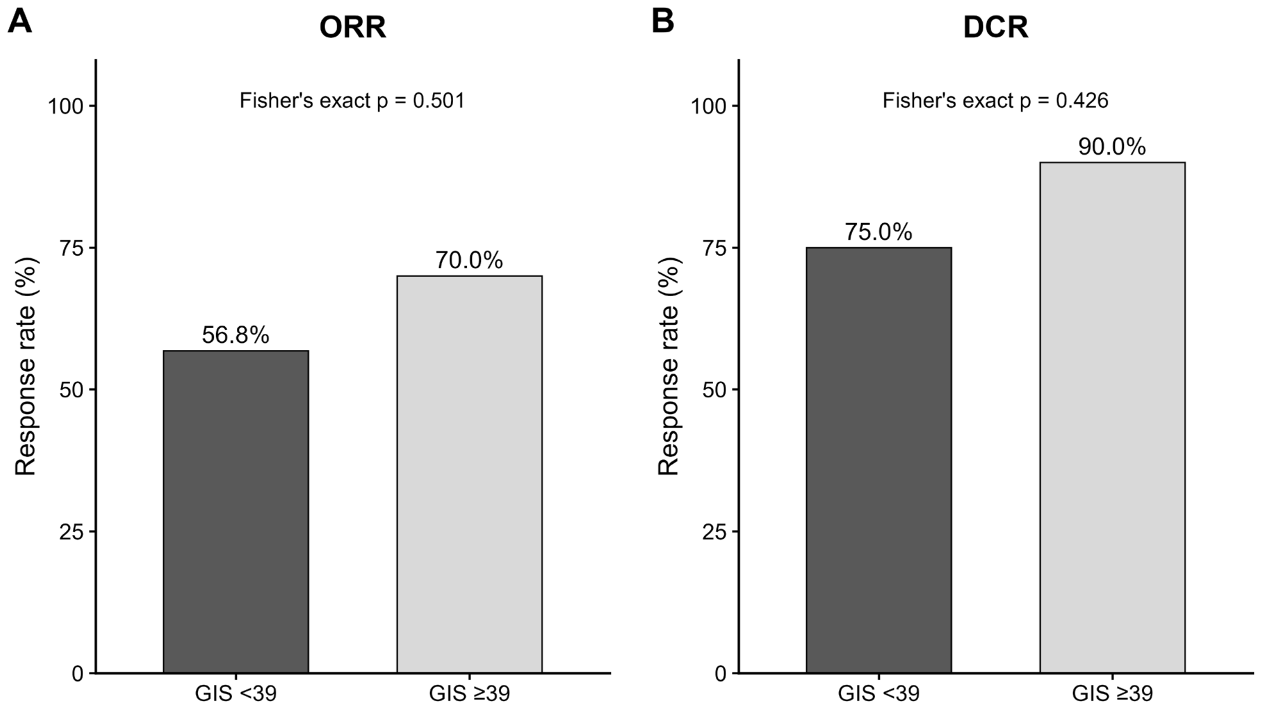

Figure S5. Radiological response according to GIS status in the first-line platinum cohort. (A) The objective response rate, defined as complete or partial response versus stable or progressive disease, was higher in tumors with GIS ≥39 than in tumors with GIS <39 (70.0% vs. 56.8%; Fisher's exact p = 0.501). (B) The disease-control rate, defined as complete response, partial response, or stable disease versus progressive disease, was also higher in the GIS ≥39 subgroup (90.0% vs. 75.0%; Fisher's exact p = 0.426).

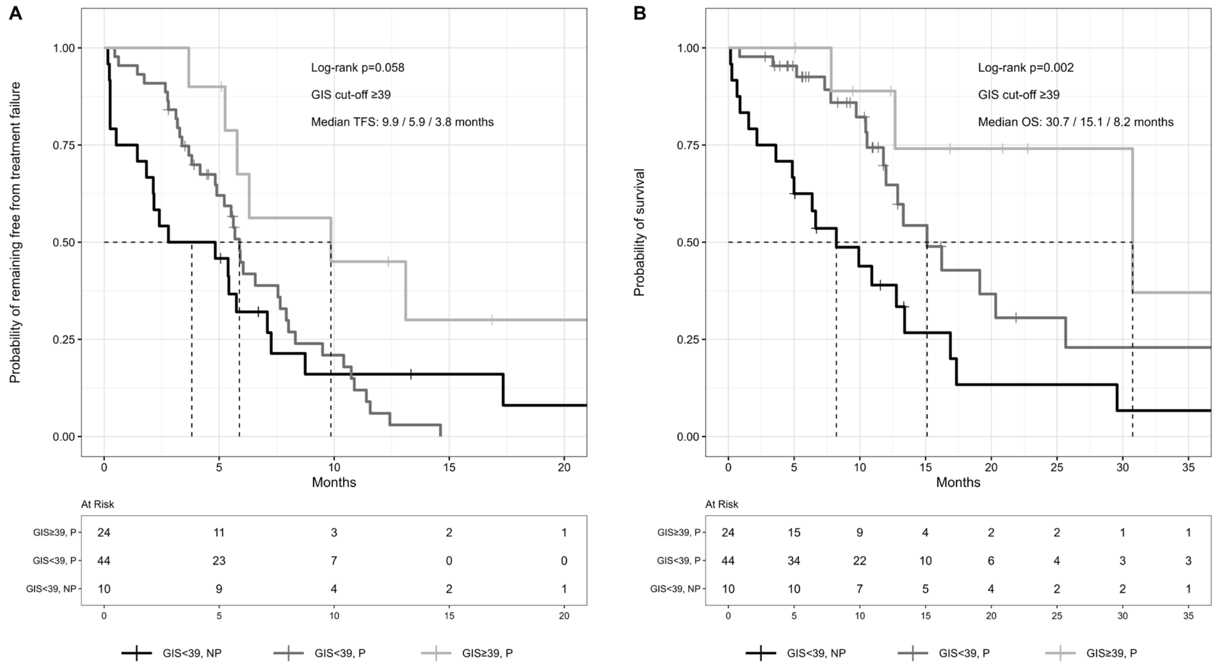

Figure S6. Three-group analyses of TFS and OS according to GIS status and platinum exposure. (A) Kaplan–Meier analysis of time to failure of the first-line strategy (TFS) comparing GIS <39 non-platinum-treated, GIS <39 platinum-treated, and GIS ≥39 platinum-treated patients. (B) Kaplan–Meier analysis of overall survival (OS) across the same three groups.

Table S1. Patient characteristics of the GIS-high subgroup.

Patient ID	Age at diagnosis	Sex	GIS	TMB (mut/Mb)	Tumor cell content (%)	Sample source	Mutation pattern	*BRCA* / HRR alteration	Co-alterations	TFS (months)	OS (months)
123	53.1	Male	60	7.9	60	Primary tumor	*BRCA* mutation	*BRCA2* c.1053dupA (41.9%)	*ERBB2* c.929C>T (31.5%); *TP53* c.626_627delGA (46.5%); *CDKN2A* c.238C>T (63.1%)	5.3	9.5
47	60.3	Female	58	5.5	50	Liver metastasis	*BRCA* mutation	*BRCA2* c.7762_7766dupATACC (69.1%)	*KRAS* c.34G>C (16.6%); *SMAD4* c.939_945delCATTTCC (13.3%); *TP53* c.586C>T (22.5%); *MAP3K4* c.2582_2595dup (5.6%); *PTPRS* c.4421_4429delinsTC (7.4%); *RICTOR* c.4166_4208delinsAGTTAT (8.0%); *ERCC1* c.320_321insAT (4.1%); *GSK3B* c.221_250del (9.8%)	3.7	48.8
38	52.7	Female	54	7.9	80	Liver metastasis	*BRCA* mutation	*BRCA2* c.3785C>A (69.6%)	*KRAS* c.35G>T (38.8%); *SMAD4* c.699_728delinsGCACA (49.0%); *TP53* c.586C>T (68.9%)	6.3	12.7
43	47.1	Male	54	5.5	80	Primary tumor	*BRCA* mutation	*BRCA2* c.2312T>G (56.3%)	*TP53* c.892G>T (5.3%); *LATS1* c.160_176del (18.3%); *SMAD4* c.988G>A (13.1%)	12.4	12.4
117	64.3	Male	52	5.5	30	Primary tumor	*BRCA* mutation	*BRCA1* c.3018_3021delTTCA (46.9%)	*KRAS* c.35G>T (15.6%); *TP53* c.764T>A (17.2%); *CDKN2A* c.213C>A (23.8%); *ASXL1* c.2868_2869insG (8.0%)	16.9	16.9
126	62.4	Male	44	4.0	60	Primary tumor	*BRCA* mutation	*BRCA2* c.5796_5797delTA	*RB1* c.1237delG (82.4%); *CREBBP* c.4134-1G>A (51.8%)	5.1	5.1
9	72.3	Female	39	7.1	50	Primary tumor	*BRCA* mutation	*BRCA2* c.7762_7766dupATACC (78.4%)	*KRAS* c.35G>T (28.9%); *TP53* c.485_491dupTCTACAA (44.4%); *RNF43* c.337C>T (36.2%)	22.8	22.8
14	72.1	Male	46	4.0	80	Primary tumor	Non-*BRCA* HRR mutation	*ATM* c.4236+1G>C (43.7%)	*KRAS* c.35G>T (29.5%)	9.9	20.9
120	66.1	Female	54	7.8	60	Primary tumor	Other mutation	-	*KRAS* c.34G>C (48.7%); *TP53* c.817C>T (53.3%); *MUTYH* c.1187G>A (48.2%)	13.1	30.7
100	60.2	Male	50	6.3	70	Not specified	Other mutation	-	*KRAS* c.35G>A (36.4%); *TP53* c.318C>G (26.3%); *CDKN2A* c.224_230dupCGCCAC (27.2%); *ZFHX3* c.11012delA (15.3%)	5.8	7.8

Abbreviations: GIS, Genomic Instability Score; HRR, homologous recombination repair; TMB, tumor mutational burden; mut/Mb, mutations per megabase; VAF, variant allele frequency; TFS, time to failure of the first-line strategy; OS, overall survival. Values in parentheses after variants indicate VAF.

Table S2. Baseline characteristics and subsequent treatment exposure in the first-line platinum outcome cohort according to Genomic Instability Score status.

Characteristic	Total first-line platinum (N = 54)	GIS <39 (n = 44)	GIS ≥39 (n = 10)	p-value*
**Continuous variables, median (IQR)**				
Age at therapy initiation, years	62.5 (55.2–66.0)	63.0 (55.8–66.2)	61.0 (54.8–65.5)	0.867
Tumor mutational burden (mut/Mb)	3.5 (2.4–5.5)	3.1 (2.2–4.7)	5.9 (5.5–7.6)	<0.001
Tumor cell content (%)	50.0 (36.2–60.0)	50.0 (35.0–60.0)	60.0 (52.5–77.5)	0.049
Genomic Instability Score (GIS)	20.0 (14.2–35.5)	17.5 (14.0–24.2)	53.0 (47.0–54.0)	—
**Categorical variables, n (%)**				
**ECOG performance status**				
– 0	31 (57.4%)	24 (54.5%)	7 (70.0%)	
– 1	21 (38.9%)	18 (40.9%)	3 (30.0%)	
– 2	2 (3.7%)	2 (4.5%)	0 (0.0%)	0.816
**Sex**				
– Female	21 (38.9%)	17 (38.6%)	4 (40.0%)	
– Male	33 (61.1%)	27 (61.4%)	6 (60.0%)	1.000
**Ethnicity**				
– Caucasian	46 (85.2%)	38 (86.4%)	8 (80.0%)	
– Other	8 (14.8%)	6 (13.6%)	2 (20.0%)	0.632
**MSI status**				
– MSS	54 (100.0%)	44 (100.0%)	10 (100.0%)	—
**HRR genes**				
BRCA1/2 mutation, n (%)	8 (14.8%)	1 (2.3%)	7 (70.0%)	<0.001
Other HRR gene mutation, n (%)	6 (11.1%)	5 (11.4%)	1 (10.0%)	1.000
Metastatic sites				

Characteristic	Total first-line platinum (N = 54)	GIS <39 (n = 44)	GIS ≥39 (n = 10)	p-value*
Lymph node metastases	24 (44.4%)	19 (43.2%)	5 (50.0%)	0.736
Lung metastases	7 (13.0%)	6 (13.6%)	1 (10.0%)	1.000
Bone metastases	3 (5.6%)	3 (6.8%)	0 (0.0%)	1.000
Liver metastases	31 (57.4%)	23 (52.3%)	8 (80.0%)	0.161
Peritoneal carcinomatosis	10 (18.5%)	9 (20.5%)	1 (10.0%)	0.667
Other metastases	3 (5.6%)	3 (6.8%)	0 (0.0%)	1.000
Number of metastatic sites, median (IQR)	1.0 (1.0–2.0)	1.0 (1.0–2.0)	1.5 (1.0–2.0)	0.620
**Subsequent treatment exposure†**				
Number of treatment lines, median (IQR)	2.0 (1.0–3.0)	2.0 (1.0–3.0)	1.5 (1.0–2.0)	0.214
Received ≥2 treatment lines, n (%)	37 (68.5%)	32 (72.7%)	5 (50.0%)	0.257
**Distribution among patients receiving ≥2 lines**				
– Exactly 2 lines	21/37 (56.8%)	18/32 (56.3%)	3/5 (60.0%)	
– ≥3 lines	16/37 (43.2%)	14/32 (43.8%)	2/5 (40.0%)	1.000

* p-values compare GIS <39 and GIS ≥39 using the Mann–Whitney U test for continuous variables and Fisher's exact test for categorical variables; the Fisher–Freeman–Halton extension was used for ECOG performance status. GIS was not tested because it defines the comparison groups. † Subsequent treatment exposure is a post-baseline variable and was included only in exploratory multivariable TFS analyses. Percentages for exactly 2 versus ≥3 lines are calculated among patients who received at least two treatment lines.

Abbreviations: ECOG, Eastern Cooperative Oncology Group; GIS, Genomic Instability Score; HRR, homologous recombination repair; IQR, interquartile range; MSI, microsatellite instability; MSS, microsatellite stable; mut/Mb, mutations per megabase; TFS, time to failure of the first-line strategy.

Supplementary File S1. Completed ESMO-GROW checklist. The checklist was completed according to the ESMO Guidance for Reporting Oncology real-World evidence (GROW) [35,36].

ESMO GROW

GUIDANCE FOR REPORTING ONCOLOGY REAL-WORLD EVIDENCE

ESMO-GROW Checklist for Authors and Reviewers

Recommendations	Yes, fully reported	Yes, partially reported	Not reported	Not applicable
**4. Results**				
4.1: Provide number of cases excluded or nonparticipating and reasons at each stage of sample selection, as well as numbers lost to follow-up. Compare the cases excluded with those included in the analyses. Illustrate this with a flowchart	●	○	○	○
4.2: Describe the baseline characteristics of the cases included (e.g. clinico-demographic and tumour characteristics). The baseline characteristics of different groups under analysis should be compared, if applicable	●	○	○	○
4.3: Report the results of the primary analysis of study outcomes. Briefly describe the results of exploratory analyses if relevant (prespecified and/or *post hoc*). Provide details of how readers can access the full results	●	○	○	○
**5. Discussion and conclusions**				
Discussion				
5.1: Summarise the core results that address the primary research question(s) and objectively discuss the data in relation to the best available evidence on the topic. Avoid a convenient selection of literature to support a point	●	○	○	○
5.2: Discuss the strengths and limitations of the current study, including the main biases, how the strategies applied contributed to bias avoidance or mitigation, and, if applicable, in which direction the authors estimate that residual bias may influence the core results of the study	○	●	○	○
5.3: Discuss the generalisability of the study results and their potential implications for clinical practice, health policies or public health and for the generation of hypotheses for future research	●	○	○	○
Conclusions				
5.4: Provide a balanced summary of core results relating to the primary research question and the main implications for clinical practice, health policies and/or public health. Suggest further research considering the remaining unmet needs and limitations from the reported study	●	○	○	○
**6. Final considerations**				
6.1: Specify all relevant study sponsorship(s) as well as direct and/or indirect or in-kind funding	●	○	○	○
6.2: Specify all relevant acknowledgements, author disclosures, individual contributions and other final considerations as per journal regulations	●	○	○	○

Notes:

To access full manuscripts and for citations, please consider the following references and links:

- Castelo-Branco L et al. "ESMO Guidance for Reporting Oncology real-World evidence (GROW)". Ann Oncol 2023; 34: 10.1016/j.annonc.2023.10.001
- Castelo-Branco L et al. "ESMO Guidance for Reporting Oncology real-World evidence (GROW)". ESMO Real World Data & Digital Oncol 2023; 1: 10.1016/j.esmorw.2023.10.001

ESMO GOOD SCIENCE BETTER MEDICINE BEST PRACTICE

ESMO GROW
GUIDANCE FOR REPORTING ONCOLOGY REAL-WORLD EVIDENCE

ESMO-GROW Checklist for Authors and Reviewers

This checklist integrates all ESMO-GROW recommendation and could be used by authors and reviewers when assessing the reporting standards of a real-world evidence study in Oncology.

For the the ESMO-GROW checklist, the following criteria are considered:
"Yes, fully reported" – The recommendation is adequately considered.
"Yes, partially reported" – The recommendation is considered, but some important details are missing.
"Not reported" – The recommendation is applicable for the case, but it was not considered.
"Not applicable" – The recommendation is not applicable for this study.

Name of Author/Reviewer: Justus Leinhos **Date:** 02.07.2026

Title of Manuscript or Identifier: Optimized Genomic Instability Score (GIS) Cutoff for Predicting First-Line Platinum Sensitivity in Non-Curative Pancreatic Cancer: Analysis from a Multicenter Real-World Cohort

Recommendations	Yes, fully reported	Yes, partially reported	Not reported	Not applicable
**1. Title**				
1.1: Concisely include relevant key terms referring to the study type, study population, objectives, data sources and outcomes, depending on the study. Consider including the terms 'real-world' or 'observational'	●	○	○	○
**2. Introduction**				
2.1: Explain the scientific rationale for the research question(s), providing concise background information on previous core evidence from systematic reviews, meta-analyses, clinical trials and/or real-world evidence studies	●	○	○	○
2.2: Identify the gaps in evidence and explain why and how they can be suitably addressed by real-world evidence research. Specify the new evidence that is expected from the current study	●	○	○	○
2.3: Briefly introduce the aim(s) of the study	●	○	○	○
**3. Methods**				
Study objective(s), design, data sources and variables				
3.1: Provide the study research question(s) including a description of the patients or the object under study and the target outcome(s)	●	○	○	○
3.2: Provide the study objective(s) and consider classifying the type of research as descriptive and/or analytical (explanatory or predictive)	●	○	○	○
3.3: Provide relevant information to describe and classify the study design used to address the research question	●	○	○	○
3.4: Give a clear definition of the eligibility criteria used to select the patients or objects under study, particularly regarding cancer-related aspects	●	○	○	○
3.5: Report the specific type and purpose of real-world data source(s) used, providing a detailed description and the reason(s) why the source was considered appropriate for the study objectives	○	●	○	○
3.6: When multiple real-world data sources are used, provide details on interoperability, including identification of duplicated cases or data linkage from separate databases	○	●	○	○
3.7: Provide details and timings of source and study data management. Consider specifying methods of raw data collection, updates and completeness, data extraction, cleaning and/or quality controls and validation	○	●	○	○
3.8: Provide core details on database and/or study registration, governance, ownership, metadata and full data accessibility in the main text or supplementary material	○	●	○	○
3.9: Identify the data source of each core variable, its definition, if the variable was derived or coded, and describe how the derivation or coding was conducted and validated.	○	●	○	○
3.10: Specify the time points of core variables in relation to the cancer disease trajectory	○	●	○	○
3.11: Provide a complete list of core variables included in the study. Variables can be grouped as baseline characteristics, exposure and outcomes or endpoints	●	○	○	○
3.12: For biomarker-related studies, provide details on biomarker description, timing, and methods of assessment and analytical validation	●	○	○	○
Statistical analysis and artificial intelligence methods				
3.13: Summarise the main aspects of the statistical analysis	●	○	○	○
3.14: When applicable, provide details on the pre-planned sample size requirements and power of the study	○	○	○	●
3.15: Specify the pre-planned strategies to identify and mitigate the main sources of bias	○	●	○	○
3.16: Clearly distinguish prespecified from *post hoc* analyses, especially for subgroup analyses	○	●	○	○
3.17: Provide information on internal and external validity, as well as any sensitivity analyses	○	●	○	○
3.18: For analytical studies, the full version of the statistical analysis plan should be provided in the supplementary material, including a brief explanation of any amendments	○	●	○	○
3.19: When applicable, specify which machine learning, deep learning or alternative artificial intelligence method has been used	○	○	○	●
3.20: When reporting real-world data analysis with artificial intelligence (e.g. machine learning and deep learning) algorithms, include comprehensive aspects on data pre-processing techniques, feature engineering strategies and model development	○	○	○	●
3.21: Address the artificial intelligence model explainability and interpretability, and present the plan for integration into clinical practice, if applicable	○	○	○	●
3.22: When applicable, briefly describe the multidisciplinary team required for the study and explain how these needs were met	○	○	○	●

References

35. Castelo-Branco, L.; Pellat, A.; Martins-Branco, D.; Valachis, A.; Derksen, J.W.G.; Suijkerbuijk, K.P.M.; Dafni, U.; Dellaporta, T.; Vogel, A.; Prelaj, A.; et al. ESMO Guidance for Reporting Oncology real-World evidence (GROW). Ann. Oncol. 2023, 34, 1097–1112. https://doi.org/10.1016/j.annonc.2023.10.001

36. Castelo-Branco, L.; Pellat, A.; Martins-Branco, D.; Valachis, A.; Derksen, J.W.G.; Suijkerbuijk, K.P.M.; Dafni, U.; Dellaporta, T.; Vogel, A.; Prelaj, A.; et al. ESMO Guidance for Reporting Oncology real-World evidence (GROW). ESMO Real World Data Digit. Oncol. 2023, 1, 100003. https://doi.org/10.1016/j.esmorw.2023.10.001
